# Aerobic and resistance exercise improves Reynolds risk score in overweight or obese breast cancer survivors

**DOI:** 10.1186/s40959-020-00084-6

**Published:** 2020-11-24

**Authors:** Kyuwan Lee, Nathalie Sami, Debu Tripathy, Wendy Demark-Wahnefried, Mary K. Norris, Kerry S. Courneya, Christina M. Dieli-Conwright

**Affiliations:** 1 Department of Population Sciences, Beckman Research Institute, City of Hope, Duarte, CA 91010 USA; 2 Department of Medicine, Keck School of Medicine, University of Southern California, Los Angeles, CA 90033 USA; 3 Department of Breast Medical Oncology, The University of Texas MD Anderson Cancer Center, Houston, TX 77030 USA; 4 Department of Nutrition Sciences, University of Alabama at Birmingham, Birmingham, AL 35294 USA; 5 Division of Population Sciences, Department of Medical Oncology, Dana-Farber Cancer Institute, Harvard Medical School, 375 Longwood Avenue, Boston, MA 02215 USA; 6 Faculty of Kinesiology, Sport, and Recreation, University of Alberta, Edmonton, Alberta T6G 2H9 Canada

**Keywords:** Exercise, Cardiovascular disease, Cancer

## Abstract

**Background:**

Breast cancer survivors have double the risk of mortality from cardiovascular disease than age-matched women without a cancer history. Reynolds risk score (RRS) is a validated algorithm for the assessment of cardiovascular disease risk. This secondary analysis sought to examine the effects of a 16-week aerobic and resistance exercise intervention on RRS in overweight or obese breast cancer survivors.

**Methods and results:**

One hundred overweight or obese (BMI > 25 kg/m^2^) breast cancer survivors were randomized to exercise or usual care. The exercise group underwent aerobic and resistance exercise sessions for 16 weeks. RRS was calculated using a validated equation. Group differences in mean change for RRS were evaluated using repeated-measures analyses of variance. Post-intervention, RRS was significantly reduced (7.9 ± 0.9% to 1.0 ± 0.5%; *p* < 0.001) in the exercise group compared to a significant increase (9.0 ± 0.8% to 11.6 ± 1.2%; *p* = 0.002%) in the usual care group (*p* < 0.01). RRS was significantly reduced in exercise vs usual care (between group difference, − 10.6; 95% CI, − 16.3 to − 7.4; *p* < 0.001).

**Conclusion:**

A 16-week aerobic and resistance exercise intervention is an effective approach to reduce the risk of cardiovascular disease in breast cancer survivors. Exercise during cancer survivorship should be considered to reduce the risk for cardiovascular disease risk in overweight women breast cancer survivors.

**Trial registration:**

ClinicalTrials.gov: NCT01140282. Registered 9 June 2010

## Background

Breast cancer survivors have approximately double the risk of mortality from cardiovascular disease than age-matched women without a cancer history [1]. Framingham risk score (FRS) has been used to estimate the 10-year risk of cardiovascular disease in breast cancer survivors [2]. However, the accuracy of FRS has been limited due to the absence of inflammatory markers, glycated hemoglobin (HbA1c) and family history of cardiovascular disease in the prediction algorithm [3]. Reynolds risk score (RRS) was developed in 2007 for use in women and men for the assessment of cardiovascular disease which incorporates high sensitivity C-Reactive Protein (hsCRP), HbA1c, and parental history of myocardial infarction, along with traditional biomarkers including total cholesterol (TC), high-density lipoprotein cholesterol (HDL-C), systolic blood pressure (SBP), and presence of diabetes. The algorithm was validated in a large multi-ethnic Women’s Health Initiative Observational Cohort [4].

We previously reported significant improvements in FRS following a 16-week resistance and aerobic exercise intervention resulting from the improvements in SBP, HDL-C, and TC in overweight or obese women with breast cancer [5]. Here, we report the findings of an unplanned secondary analysis of a randomized controlled trial comparing a progressive aerobic and resistance exercise intervention versus usual care on baseline to 4-month changes in overweight or obese women with breast cancer. Detailed methods [6], and primary outcomes related to metabolic syndrome were published previously [7]. We hypothesized that a 16-week progressive aerobic and resistance exercise intervention would reduce RRS in sedentary, overweight or obese patients with early stage breast cancer compared to usual care.

## Methods

One hundred eligible women with breast cancer were recruited between August 1, 2012 and December 31, 2016. The protocol and informed consent were approved by the institutional review board (HS-12-00141) and participants gave written informed consent. Participants were < 6 months post-treatment for stage I-III female breast cancer and were nonsmokers, sedentary (< 60 min of structured exercise/week), with body mass index (BMI) ≥25.0 kg/m^2^ or body fat > 30% and waist circumference > 88 cm.

RRS was calculated using gender specific validated methodology [4], at baseline and week 17. The following data were collected in order to calculate RRS: age, SBP, hsCRP, TC, HDL-C, HbA1c, current smoking status, and parental history of myocardial infarction. Due to the primary study design, current smokers were ineligible and therefore smoking status did not vary by patient. Age and parental history of myocardial infarction were abstracted from medical records. SBP was measured three times using the arm contralateral to the affected breast with an automated sphygmomanometer after five minutes of quiet sitting, (Welch Allyn, Skaneateles Falls, New York). Plasma and serum biomarkers were analyzed from fasting (≥12 h) blood samples that were stored at − 80 °C until batch analysis at study completion. The following RRS equation was used to calculate 10-year cardiovascular disease risk (%) = [1–0.98634(exp[B − 22.325])] × 100% where B = 0.0799 × age + 3.137 × natural logarithm (SBP) + 0.180 × natural logarithm (high-sensitivity C-reactive protein) + 1.382 × natural logarithm (TC) − 1.172 × natural logarithm (HDL-C) + 0.134 × HbA1c (%) (if diabetic) + 0.818 (if current smoker) + 0.438 (if family history of premature myocardial infarction).

The 16-week exercise intervention was designed based on American College of Sports Medicine/American Cancer Society exercise guidelines for cancer survivors (≥150 min of aerobic exercise and 2–3 days of resistance exercise training/week) [8, 9]. Women participated in thrice weekly supervised one-on-one exercise sessions for 16 weeks. Days one and three endorsed resistance and aerobic exercise of approximately 80 min. Day two included approximately 50 min of aerobic exercise. Aerobic exercise included treadmill walking, rowing machine, or stationary cycling. Heart rate was monitored throughout the aerobic exercise sessions to maintain the heart rate at 65 to 80% maximum heart rate. Resistance exercise sessions included leg press, leg flexion, leg extension, chest press, seated row, bicep curls, and triceps pulldowns. Initial resistance was set at 80% of 1-repetition maximum (1-RM) for lower body exercises and 60% 1-RM for upper body exercises which were determined during baseline testing.

The sample size was based on changes in insulin with a 16-week exercise intervention among survivors of breast cancer. Enrollment of 100 women provided 80% statistical power (*p* < 0.05) to detect a 2.6-mU/mL (SD = 4.0 mU/mL) difference in mean insulin levels, assuming 20% dropout using a two-group *t* test. Within-group and between-group differences in mean change for individual outcomes measured at 16 weeks were evaluated using general linear models repeated-measures analyses of variance and mixed model repeated-measures analysis, respectively. A priori covariates with potential associations with the outcome of interest (e.g., type of treatment [chemotherapy, radiation, or both], surgery-type, medication use [e.g., anti-hypertensives and hyperglycemia medications], BMI, caloric intake, diet quality, and macronutrient distribution) were explored in models, but none modified the results. Analyses were performed using SAS (SAS/STAT User’s Guide, Version 9.4; SAS Institute, Cary, NC).

## Results

Baseline characteristics are reported elsewhere [7], and there were no significant differences between the two groups. The women were Hispanic white (55%) or non-Hispanic (45%) and on average, 53.5 ± 10.4 years old, 6.2 ± 2.1 months from diagnosis, with a BMI of 33.5 ± 5.5 kg/m^2^. Women who underwent chemotherapy and were potentially exposed to cardiotoxic therapies and represented 10% of the total sample. Those with left sided breast irradiation included 22 participants in the exercise group and 19 in the control group. No difference in results were found when stratified by this variable. Exercise intervention compliance was 95%, a strong indicator that the exercise program was well tolerated. No adverse events occurred during the study. Following the 16-week intervention period, RRS was significantly reduced (7.9 ± 0.9% to 1.0 ± 0.5%; *p* < 0.001) in the exercise group compared to the usual care group (Table 1). The significant reduction of RRS resulted from improvements in hsCRP (3.4 ± 0.6 to 2.3 ± 0.3 mg/L; p < 0.001), HbA1c (7.5 ± 0.9 to 2.9 ± 0.2%; p < 0.001), and previously reported values of SBP (132.9 ± 13.0 to 120.7 ± 9.5 mmHg; p < 0.001), HDL-C (43.1 ± 6.6 to 64.7 ± 7.8 mg/dL) and TC (196.5 ± 53.4 to 157.5 ± 37.1 mg/dL) [7] in the exercise group compared to the usual care group. In contrast, there was a significant increase in RRS (9.0 ± 0.8 to 11.6 ± 1.2%; *p* = 0.002) in the usual care group, resulting from slight, non-statistically significant increases in HbA1c (7.5 ± 0.8 to 7.9 ± 0.8; *p* = 0.28), and previously reported values of TC (194.4 ± 48.9 to 210.4 ± 52.2; *p* = 0.31), HDL-C (41.0 ± 4.3to 39.9 ± 4.0; *p* = 0.45), SBP (133.7 ± 9.7to 135.9 ± 9.8; *p* = 0.22), hsCRP (3.7 ± 0.3 to 4.2 ± 0.4; *p* = 0.54) [7].

**Table 1:** Comparison of Reynolds risk score variables between exercise and usual care groups

Variable	Baseline, mean (SD)	Post-intervention		Between Group Difference Post intervention	
	Mean (SD)	Mean (SD)	*P* *	Mean (95% CI)	*P* ^†^
**Age (yrs)**					
Exercise	52.8 (10.6); (*n* = 50)	52.8 (10.6); (*n* = 48)	–	–	–
Usual Care	53.6 (10.1); (n = 50)	53.6 (10.1); (*n* = 45)			
**Systolic blood pressure (mmHg)**					
Exercise	132.9 (13.0)	120.7 (9.5)	< 0.001*	−13.7 (− 16.5 to − 8.7)	< 0.001^†^
Usual Care	133.7 (9.7)	135.9 (9.8)	0.22		
**hsCRP (mg/L)**					
Exercise	3.4 (0.6)	2.3 (0.3)	< 0.001*	−1.9 (− 5.2 to − 0.6)	< 0.001^†^
Usual Care	3.7 (0.3)	4.2 (0.4)	0.54		
**Total Cholesterol (mg/dL)**					
Exercise	196.5 (53.4)	157.5 (37.1)	< 0.001*	−52.9 (−75.0 to −43.0)	< 0.001^†^
Usual Care	194.4 (48.9)	210.4 (52.2)	0.31		
**HDL-C (mg/dL)**					
Exercise	43.1 (6.6)	64.7 (7.8)	< 0.001*	24.4 (17.9 to 27.9)	< 0.001^†^
Usual Care	41.0 (4.3)	39.9 (4.0)	0.45		
**Hemoglobin A1C (%)**					
Exercise	7.5 (0.9)	2.9 (0.2)	< 0.001*	−4.6 (−11.2 to −1.6)	< 0.001^†^
Usual Care	7.5 (0.8)	7.9 (0.8)	0.28		
**Parent with MI before age 60 years, n (%)**					
Exercise	40 (80)	40 (80)	–	–	–
Usual Care	42 (84)	42 (84)			
**10-year Risk RRS (%)**					
Exercise	7.9 (0.9)	1.0 (0.5)	< 0.001*	−10.6 (−16.3 to −7.4)	< 0.001^†^
Usual Care	9.0 (0.8)	11.6 (1.2)	0.002*		

*P value for repeated-measures ANOVA comparing changes in the exercise group from baseline to post-intervention, and in the usual care group from baseline to post-intervention

^†^ *P* value for mixed model analysis comparing changes between the exercise and usual care group from baseline to post-intervention

Age and parent history of MI before 60 years calculated as unchanged during the 16-week intervention period

## Discussion

This secondary and unplanned analysis of supervised 16-week aerobic and resistance exercise intervention originally designed to improve metabolic syndrome, also led to clinically significant reductions in RRS for 10-year risk of developing cardiovascular disease among sedentary, overweight/obese patients with early stage breast cancer. While FRS includes age, SBP, smoking, diabetes, and total and HDL-C in a prediction algorithm, cardiovascular disease risk is also associated with family history, markers of inflammation such as hsCRP, and HbA1c among individuals without known cardiovascular diseases [10–12]. Given that an overweight and obese status at the time of diagnosis or weight gain after diagnosis of breast cancer is associated with elevated levels of inflammatory markers (i.e. hsCRP) [13], and metabolic disturbances (i.e. HbA1C) [14], RRS may be useful, and perhaps a more indicative measure to estimate the future risk of developing cardiovascular disease in breast cancer survivors.

This is the first randomized clinical trial of exercise training in women with early stage breast cancer to demonstrate the benefits of an exercise intervention on RRS in any clinical populations. While we previously reported aerobic and resistance exercise improves FRS [5], our findings were limited because inflammatory and diabetes-related status were not accounted for in the FRS. Cardiovascular disease risk assessed by RRS may address these limitations, and RRS was significantly reduced by our intervention. As HbA1c and hsCRP are associated with obesity [10, 11], and our overweight/obese participants significantly lost body fat following 16 weeks [7], the large effects of exercise may have occurred by regulating adipose tissue yet further mechanisms need to be identified at the tissue level. In addition, our study was limited to overweight and obese women thus future studies should include regular weight women (BMI < 25) to determine whether RRS would have a similar decrease in non-overweight/obese breast cancer survivors. Lastly, RRS increased in the usual care group following the 16-week study period demonstrating increased risk of future cardiovascular disease yet no change in FRS was previously found, possibly due to the improved sensitivity of RRS algorithm as reported by Cook et al. (2012) [15].

In conclusion, a 16-week resistance and aerobic exercise intervention decreased the 10-year risk of developing cardiovascular disease assessed by RRS by decreasing hsCRP, HbA1c, SBP, TC and increasing HDL-C. Overweight/obese women breast cancer survivors may consider performing regular aerobic and resistance exercise to reduce future cardiovascular disease during cancer survivorship.

## Data Availability

The datasets used and/or analyzed during the current study are available from the corresponding author on reasonable request.

## Abbreviations

RRS: Reynolds risk score
BMI: Body mass index
FRS: Framingham risk score
HbA1c: Glycated hemoglobin
hsCRP: C-reactive protein
TC: total cholesterol
HDL-C: High-density lipoprotein cholesterol
SBP: Systolic Blood Pressure
1-RM: 1-repetition maximum
SD: Standard deviation
